# EPAC in Vascular Smooth Muscle Cells

**DOI:** 10.3390/ijms21145160

**Published:** 2020-07-21

**Authors:** Nadine Wehbe, Suzanne Awni Nasser, Yusra Al-Dhaheri, Rabah Iratni, Alessandra Bitto, Ahmed F. El-Yazbi, Adnan Badran, Firas Kobeissy, Elias Baydoun, Ali H. Eid

**Affiliations:** 1 Department of Biology, American University of Beirut, P.O. Box 11-0236, Beirut, Lebanon; nww04@mail.aub.edu (N.W.); eliasbay@aub.edu.lb (E.B.); 2 Department of Pharmacology and Therapeutics, Beirut Arab University, P.O. Box 11-5020, Beirut, Lebanon; san413@bau.edu.lb; 3 Department of Biology, United Arab Emirates University, P.O. Box 15551, Al-Ain, UAE; yusra.aldhaheri@uaeu.ac.ae (Y.A.-D.); r_iratni@uaeu.ac.ae (R.I.); 4 Department of Clinical and Experimental Medicine, University of Messina, 98125 Messina, Italy; abitto@unime.it; 5 Department of Pharmacology and Toxicology, American University of Beirut, P.O. Box 11-0236, Beirut, Lebanon; ae88@aub.edu.lb; 6 Department of Pharmacology and Toxicology, Alexandria University, 21526 Alexandria, Egypt; 7 Department of Nutrition, University of Petra, P.O. Box 961343, Amman 11196, Jordan; abadran@uop.edu.jo; 8 Department of Biochemistry and Molecular Genetics, American University of Beirut, P.O. Box 11-0236, Beirut, Lebanon; firasko@gmail.com; 9 Department of Biomedical Sciences, Qatar University, P.O. Box 2713, Doha, Qatar

**Keywords:** EPAC, vascular smooth muscle cells, ROS, cAMP, phenotypic switch, cardiovascular disease

## Abstract

Vascular smooth muscle cells (VSMCs) are major components of blood vessels. They regulate physiological functions, such as vascular tone and blood flow. Under pathological conditions, VSMCs undergo a remodeling process known as phenotypic switching. During this process, VSMCs lose their contractility and acquire a synthetic phenotype, where they over-proliferate and migrate from the tunica media to the tunica interna, contributing to the occlusion of blood vessels. Since their discovery as effector proteins of cyclic adenosine 3′,5′-monophosphate (cAMP), exchange proteins activated by cAMP (EPACs) have been shown to play vital roles in a plethora of pathways in different cell systems. While extensive research to identify the role of EPAC in the vasculature has been conducted, much remains to be explored to resolve the reported discordance in EPAC’s effects. In this paper, we review the role of EPAC in VSMCs, namely its regulation of the vascular tone and phenotypic switching, with the likely involvement of reactive oxygen species (ROS) in the interplay between EPAC and its targets/effectors.

## 1. Introduction

Cyclic adenosine 3′,5′-monophosphate (cAMP) is one of the most studied second messengers that play a critical role in intracellular signaling transduction. It controls a wide variety of cellular responses including cell proliferation, migration, differentiation and apoptosis. Particularly in the cardiovasculature, cAMP’s role has been overwhelmingly documented [1,2,3,4,5,6,7,8,9,10,11]. cAMP is generated from ATP by the action of adenylyl cyclase (AC) isoforms, either membrane-bound or soluble [12,13]. The intracellular level of cAMP depends on its production by ACs and its degradation by cAMP phosphodiesterases (PDEs), which catalyze the hydrolysis of cAMP into 5′-adenosine monophosphate (5′-AMP) [14]. In addition to ACs and PDEs, the intracellular level of cAMP is regulated by A-kinase anchoring proteins (AKAPs), scaffolding proteins that sequester cAMP and its relevant signaling components into defined subcellular compartments [15]. This compartmentalization helps in sustaining localized pools of cAMP to effectively modulate the cellular actions of this second messenger [16,17].

A decade after the discovery of cAMP, protein kinase A (PKA) was identified as the downstream effector mediating cAMP signaling [18]. Later in 1998, two independent research groups discovered a novel cAMP effector family, currently known as exchange proteins activated by cAMP (EPACs) [19,20]. EPAC proteins, EPAC1 and EPAC2, act as guanine nucleotide exchange factors (GEFs) for the small Ras-like GTPases, Rap1 and Rap2. Although EPAC and PKA may act independently, they are often associated with the same biological process wherein they could mediate synergistic or opposite effects. The discovery of EPAC is relatively new; however, it has been shown to significantly modulate a plethora of pathways in different cell systems. It can control key cellular processes, such as cell proliferation, migration and apoptosis [1,3,16,21]. Many studies have demonstrated an important role of EPAC in the cardiovascular system. In addition to its role in physiology, EPAC is a key contributor to several cardiovascular pathologies [22,23].

Vascular smooth muscle cells (VSMCs) are major components of blood vessels and are located in the tunica media, the middle layer of a vessel wall. These cells are integral to the function of a blood vessel, both under physiologic and pathophysiologic conditions [3,24]. In healthy vessels, VSMCs contribute to vasotone and the regulation of blood flow. The onset of certain pathological conditions, such as atherosclerosis or hypertension, could trigger VSMCs to undergo a remodeling process known as phenotypic switching, where they lose their contractile phenotype and acquire a synthetic one [24]. VSMCs of a synthetic phenotype excessively proliferate and migrate towards the tunica intima of vessel wall. In the tunica intima, VSMCs contribute to the formation of atheroma in atherosclerosis and neointimal hyperplasia during restenosis [24,25,26]. There are some discrepancies regarding the role of EPAC in cardiovascular pathologies, where some studies report EPAC as a mediator of VSMCs phenotypic switching, while others describe a protective role.

In the present review, we aim to focus on the role of EPAC in VSMCs, mainly its involvement in the vascular tone and phenotypic switching. We provide a detailed and critical discussion of the targeted pathways and the underlying mechanisms involved in VSMC remodeling in in vitro and in vivo models of hypertension and neointimal hyperplasia.

## 2. EPAC

### 2.1. Genes and Expression

EPAC proteins, also known as cAMP-GEF proteins, comprise two isoforms, EPAC1 and EPAC2, which are encoded by two independent genes. EPAC1 is encoded by *RAPGEF3* gene in humans and is ubiquitously expressed, particularly in the heart, blood vessels and kidney [19]. On the other hand, EPAC2, encoded by *RAPGEF4* gene in humans, is prominently expressed in the brain and adrenal glands [19,20]. Alternative splicing of *RAPGEF4* gene gives rise to three EPAC2 variants, designated as EPAC2A, EPAC2B, and EPAC2C [27]. These variants differ in their structure and tissue-specific expression. While EPAC2A is broadly expressed in the brain, pituitary and pancreas [20,27], EPAC2B is detected in the adrenal gland [28] and EPAC2C is liver specific [29].

### 2.2. Structure and Activation

**Figure 1:**
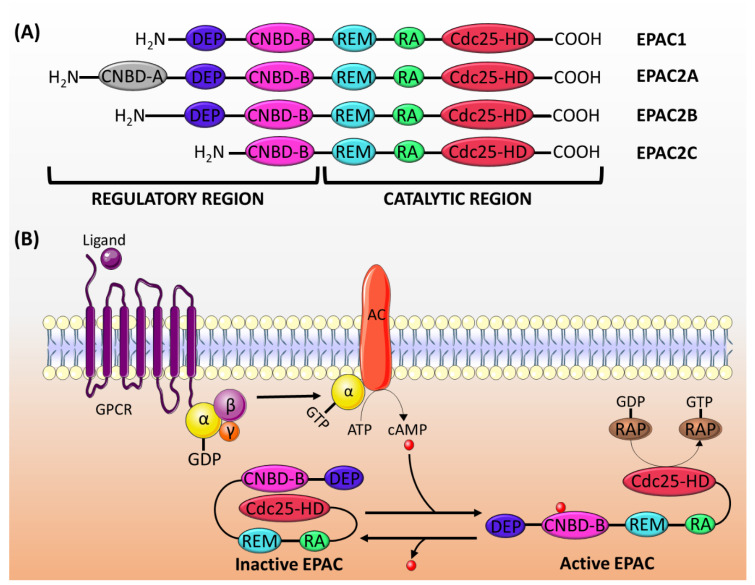
(**A**) The structure of EPAC proteins. EPAC is made up of a catalytic region and a regulatory region, each of which is divided into different domains. The catalytic region in all EPAC isoforms comprises three domains: REM, RA and Cdc25-HD. The regulatory region consists of two domains: DEP and CNBD-B. EPAC2A has an addition CNBD-A domain, whereas EPAC2C lacks the DEF domain. (**B**) The mechanism of EPAC proteins activation: the activation of adenylyl cyclase (AC) by the G_α_ subunit of Gs protein induces the production of cAMP, which binds to the CNBD-B within the regulatory region of EPAC. This binding induces a conformational change releasing the auto-inhibitory effect, and it permits the binding of Rap1/2 to the catalytic domain (Cdc25-HD) and its subsequent activation by the GEF activity of EPAC. GPCR: G-protein coupled receptor.

EPAC proteins are characterized by sequence homology to GEFs (for Ras and Rap) and to cAMP-binding sites. The structure of EPAC consists of a C-terminal catalytic region and an N-terminal regulatory region (Figure 1A). The catalytic region is similar in all isoforms and comprises a Ras-exchange motif (REM) domain, a Ras-association (RA) domain, and a cell division cycle 25 homology domain (Cdc25-HD) [16]. The REM domain, which is found in all Ras- and Rap-specific GEFs, is involved in the stabilization of the active conformation of EPAC [19,30]. On the other hand, the RA domain helps target EPAC to the membrane [31]. The Cdc25-HD is responsible for the GEF activity of EPAC [32].

The regulatory region differs among isoforms, though it generally consists of two domains: a disheveled/EGL-10/pleckstrin (DEP) domain and a cAMP-nucleotide binding-B domain (CNBD-B) [16]. The DEP domain, which is not present in EPAC2C, is necessary for the translocation of EPAC from the cytosol to the membrane [33]. The CNBD-B, as its name implies, is the binding site for cAMP. EPAC2A has an additional CNBD-A, which has a lower affinity to cAMP, and probably plays a role in the translocation of EPAC2A to the plasma membrane [34].

In its inactive state, the regulatory region of EPAC auto-inhibits its catalytic activity [19]. The cAMP binding domain acts as an inhibitory domain by binding to the Cdc25-HD and hindering the accessibility of Rap to the catalytic region. cAMP binding to CNBD induces a conformational change, which releases the auto-inhibition and promotes the binding of Rap to the Cdc25-HD motif [33] (Figure 1B).

## 3. The Role of EPAC in VSMCs

### 3.1. Vascular Tone

The regulation of the contraction and relaxation of VSMCs is critical for controlling the vasotone and blood pressure [24]. Therefore, changes in the dynamics of contraction or relaxation states may lead to pathological conditions such as hypertension. The intracellular second messenger, cAMP, modulates the vascular tone either by preventing the increase in Ca^2+^ concentration inside the cell or by reducing the Ca^2+^ sensitivity of the contractile proteins leading to the relaxation of the vessel wall [35,36].

cAMP signaling mediates vasorelaxation by the phosphorylation and inhibition of RhoA [37,38], activation of the myosin light chain phosphatase (MLCP) [39,40] as well as reduction of the inhibitory phosphorylation of myosin phosphatase-targeting subunit (MYPT1) [41,42]. The cAMP modulation of the vascular tone was attributed solely to PKA until the discovery of the novel cAMP effector, EPAC. In general, the activation of RhoA, a key regulator of the cytoskeleton, and its effector Rho kinase (ROCK), which phosphorylates and inhibits MYPT1, plays a role in mediating VSMCs contraction [43,44]. While calcium influx through CaV1.2 channels and subsequent MLCK activation is an important requirement for smooth muscle constriction [45,46], a significant body of literature highlighted the importance of RhoA-mediated calcium sensitization and actin cytoskeleton reorganization in maintaining arteriolar constriction [47,48] and providing a graded tonic response to intra-vascular pressure changes and stimulation by humoral mediators affording the auto-regulation of blood flow [49,50]. In fact, some studies have gone so far to show that the obligate dependence of vascular contractility on extracellular calcium influx is not necessarily tied to its downstream effect on MLCK and increased myosin light chain phosphorylation [51].

**Figure 2:**
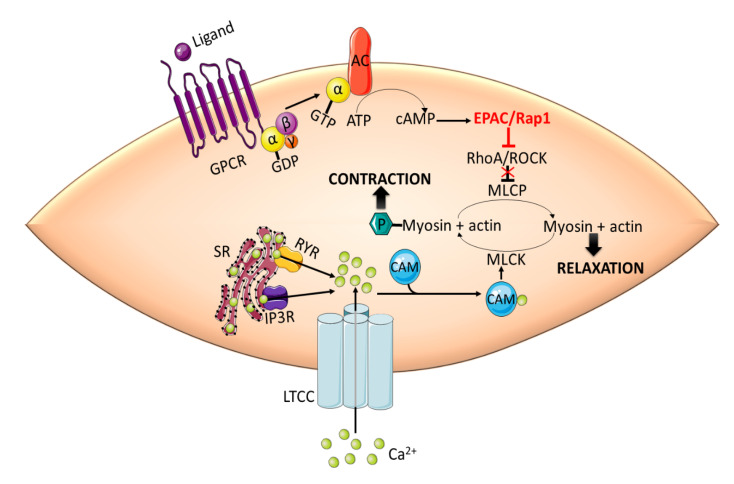
EPAC promotes vasorelaxation in vascular smooth muscle cells (VSMCs) by inhibiting RhoA/ROCK signaling. Activation of RhoA/ROCK phosphorylates MLCP and inhibits its phosphatase activity. This in turn increases the phosphorylation of MLC by MLCK, which is activated by Ca^2+^-CaM complex, and induces contraction. cAMP-mediated activation of EPAC/Rap1 releases the inhibitory effect of RhoA/ROCK on MLCP leading to the dephosphorylation of MLC and subsequent relaxation. CaM: calmodulin; GPCR: G-protein coupled receptor; IP3R: inositol 1,4,5-triphosphate receptor; LTCC: L-type calcium channel; MLC: myosin light chain; MLCK: myosin light chain kinase; RYR: ryanodine receptor; SR: sarcoplasmic reticulum.

Inhibition of phosphatase activity increases the phosphorylation of myosin regulatory light chain (RLC20) and force generation, leading to contraction [52,53]. Intracellular cAMP signaling, mediated by PKA and EPAC, induces vasorelaxation in large vessels by inhibiting RhoA/ROCK signaling. cAMP-mediated activation of EPAC results in Rap1-dependent Ca^2+^ desensitization and relaxation in rat aortic VSMCs [54]. EPAC/Rap1-mediated vasorelaxation is accomplished by the inhibition of RhoA, thus disinhibiting the MLCP activity and decreasing the phosphorylation of RLC20 (Figure 2).

Another mechanism to promote vasorelaxation is by regulating intracellular levels of Ca^2+.^ During vasorelaxation, the activation of EPAC increases the level of cytosolic Ca^2+^ by promoting its release through the ryanodine receptors of the sarcoplasmic reticulum and induces the activation of large conductance Ca^2+^-sensitive K^+^ (BKCa) channels [55]. Active BKCa channels evoke membrane hyperpolarization and limit the entry of Ca^2+^ by decreasing the activity of voltage-gated Ca^2+^ channels [56,57]. Surprisingly, EPAC-mediated increase in intracellular Ca^2+^ has been shown to inhibit ATP-sensitive potassium (KATP) channels activity through a Ca^2+^-sensitive protein phosphatase 2B (calcineurin)-dependent mechanism promoting arterial constriction [58]. Moreover, ryanodine receptor-mediated intra-cellular Ca^2+^ release has been implicated in the development of Ca^2+^ waves in VSMCs that are necessary for the maintenance of myogenic contractility in resistance arterioles [59]. Whether this effect is dependent on EPAC remains to be elucidated.

Physiologically, cAMP activates KATP channels by the virtue of its ability to stimulate PKA-dependent phosphorylation at the pore-forming and regulatory subunits of KATP, leading to vasorelaxation [60]. Interestingly, the inhibitory effect of EPAC on KATP channels has been only detected in the absence of PKA [58]. Therefore, one could speculate that EPAC promotes its inhibitory effect in pathological conditions where PKA signaling is disrupted. In rat aortic smooth muscle cells, EPAC promoted vasorelaxation by contributing to the cAMP-induced depletion of Ca^2+^ from intracellular stores and inhibition of store-operated calcium entry (SOCE). This effect of EPAC is only evident in combination with PKA activation [61]. By depleting intracellular Ca^2+^ stores, EPAC and PKA reduce Ca^2+^ availability for vasoconstriction.

In addition to its involvement in cAMP-induced endothelium-independent vasorelaxation, EPAC also contributes to vasorelaxation in the presence of an intact endothelium. Both EPAC and PKA could enhance the activity of endothelial nitric oxide synthase (eNOS), leading to an increase in nitric oxide (NO) production [55,62,63]. Contextually, knockout of Rap1, an effector of EPAC, inhibits NO-dependent vasorelaxation, resulting in heightened vasoconstriction and eventual hypertension [64]. This clearly implicates the EPAC-Rap1 signaling in endothelial dysfunction and its sequelae.

**Figure 3:**
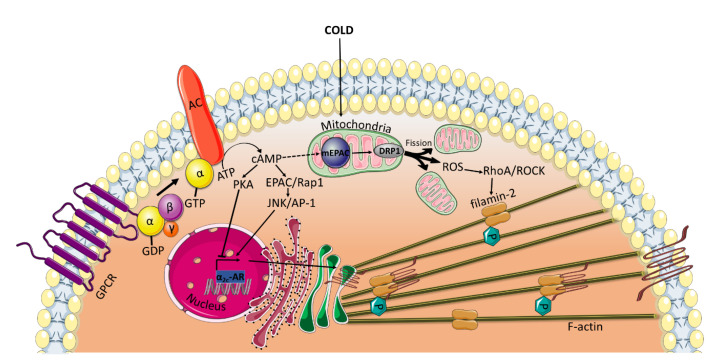
EPAC mediates cold-induced constriction in microVSMCs. Extreme cold temperatures induce the release of reactive oxygen species (ROS) form the mitochondria. Alternatively, ROS may be generated by DRP1-induced mitochondrial fission driven by mEPAC. ROS then activates RhoA/ROCK pathway, which phosphorylates filamin-2 required for the translocation of α_2C_-ARs from the transGolgi to the cell surface where they induce vasoconstriction. EPAC also plays a role in increasing the α_2C_-ARs transcription by activating JNK/AP-1 and in the translocation of the receptor by activating RhoA/ROCK. DRP1: Dynamin-related protein; mEPAC: mitochondrial EPAC.

All of the previously mentioned studies, which reveal an EPAC-mediated vasorelaxation, have been conducted using VSMCs from large vessels. Interestingly, under certain conditions, such as cold temperatures, EPAC has been found to mediate cold-induced constriction of human dermal arterioles [6,7]. In microvascular smooth muscle cells (microVSMCs) extracted from these vessels, EPAC/Rap1 increases α_2C_-adrenoreceptor (α_2C_-AR) expression by activating c-Jun N-terminal kinase (JNK)/activating protein-1 (AP-1) signaling [6,7], whereas PKA suppresses cAMP-induced activation of this receptor [4] (Figure 3). α_2C_-ARs have once been considered silent receptors, largely due to intracellular entrapment within the endoplasmic reticulum and Golgi compartment, where they are not available for their agonist [65]. However, in response to cold stimuli, α_2C_-ARs are translocated from their intracellular localization to the cell surface, where they induce vasoconstriction [4,66]. EPAC/Rap1 not only increases α_2C_-ARs expression but also contributes to the translocation of the receptor to the cell surface by activation of RhoA/ROCK signaling, reorganization of the actin cytoskeleton and increase in F-actin [6,67]. Rap1/RhoA/ROCK has been found to phosphorylate filamin-2, an actin cross-linker, at Ser2113 leading to the translocation of α_2C_-ARs [5].

The opposite positive regulatory role of EPAC/Rap1 on RhoA/ROCK in microVSMCs may be driven indirectly via mitochondrial interaction. It has been shown that the upstream regulator of EPAC, cAMP, can be transported into the mitochondria and accumulate in the matrix [68]. The transported cAMP may activate the mitochondrially localized EPAC, known to contain an NH2-terminal mitochondrial-targeting sequence essential for EPAC co-localization with the mitochondria [69]. Several lines of evidence have shown that mitochondrial EPAC is a key role player in many physiological and pathophysiological conditions. For example, EPAC contributes to modulating the phosphorylation of dynamin-related protein (DRP1) [70], which plays a critical role in mitochondrial fission/fusion dynamics [71]. Mitochondrial fission has been shown to be implicated in the generation of reactive oxygen species (ROS) [72,73], which are known to activate Rho/ROCK in VSMCs [73,74] (Figure 3). This hypothesis can be corroborated by the findings of Hui et al., who demonstrated that in a mouse carotid artery ligation model, the genetic ablation of mitochondrial EPAC mitigates DRP1-mediated mitochondrial fission and ROS production [71].

Redox signaling in mediating the differential effects of EPAC on vascular tone merits further investigation. Nonetheless, the role of EPAC in vascular tone appears to be vascular bed-specific, where it contributes to vasorelaxation in large vessels and to vasoconstriction in microvessels. This could be in part due to the absence in large vessels of α_2C_-ARs, which are known to mediate vasoconstriction [75,76].

### 3.2. Phenotypic Switching: VSMCs Proliferation and Migration

VSMC proliferation and migration are critical events involved in normal vessel growth and wound healing process as well as pathological conditions, such as atherosclerosis and neointimal hyperplasia [26]. In response to vascular injury, activated VSMCs lose their contractility and acquire a synthetic phenotype. This phenotype is characterized by increased proliferation, migration, extracellular matrix synthesis and expression of synthetic markers [24]. Therefore, both proliferation and migration must be tightly regulated to prevent the development of pathological conditions, including neointimal hyperplasia, atherosclerosis and hypertension.

There are some controversies regarding the role of cAMP and its effectors, PKA and EPAC, in cell proliferation and migration. Depending on the cell type, some studies reported an inhibitory role [77,78,79], whereas others revealed the opposite [80,81,82]. In the vasculature, cAMP is known to negatively regulate VSMC proliferation [83]. The anti-proliferative effect of cAMP was first only attributed to its effector PKA [84,85,86]. However, in rat aortic VSMCs, activation of PKA alone was insufficient to mediate cAMP-dependent cell-cycle arrest. EPAC and PKA have been shown to synergistically reduce cell proliferation by inhibiting the expression of cyclin D1 and S-phase kinase-associated protein 2 (Skp2), regulators of G1-S cell cycle phase, and attenuating mitogen-activated protein kinases (MAPKs), extracellular signal-regulated protein kinase 1/2 (ERK1/2) and JNK signaling [86]. In addition, EPAC and PKA induce a stellate morphology and cytoskeletal disruption in these cells [87]. Although Rap1 promotes cell-cycle progression in VSMCs [88], EPAC has been found to mediate its inhibitory effects in a Rap1-independent manner [88]. In another study, EPAC and PKA were also shown to synergistically reduce the expression of early growth response 1 (Egr1) [89], which has a role in cell proliferation [90,91]. By inhibiting Rac-1, EPAC and PKA promote actin-cytoskeleton remodeling and nuclear export of ERK1/2 leading to dephosphorylation of the serum response factor (SRF) co-factor Elk. This, in turn, decreases the activity of Egr1 leading to inhibition of VSMC proliferation [89].

In addition to acting in synergy with PKA, EPAC can inhibit cell proliferation in a PKA-independent manner. By downregulating the early gene nuclear receptor NR4A1, EPAC has been shown to mediate the anti-proliferative effect of adenosine A2B receptors in primary human coronary artery smooth muscle cells [92]. EPAC and PKA can also mediate opposing effects. While PKA promotes cell proliferation and hyaluronic acid production in ductus arteriosus smooth muscle cells, leading to neointimal formation [93], the treatment of these cells with the EPAC agonist, 8-pCPT-2′-O-Me-cAMP, inhibits cell proliferation and induces no change in the production of hyaluronic acid [94].

Besides its anti-proliferative effect, cAMP also plays a protective role in VSMCs by inhibiting cell migration [95,96,97]. The increase in cAMP production by beraprost, a prostacyclin analog, inhibits growth factor-induced migration of human VSMCs from saphenous veins [98]. Interestingly, therapeutically relevant concentrations of beraprost activate EPAC but not PKA. It has been reported that the EPAC/Rap1 pathway impedes RhoA signaling, preventing actin-cytoskeletal changes and subsequent migration. Notably, although high concentrations of beraprost can activate both EPAC and PKA, cell proliferation has not been detected [98]. Likewise, in rat primary thoracic aorta VSMCs, the activation of EPAC using low concentrations of an EPAC analog inhibits cell migration. EPAC mediates its inhibition via the pharmacological blockade of multiple kinase signals, such as MAPK, phosphoinositide 3-kinase (PI3K) and Rac, and by increasing paxillin and modulating focal adhesion proteins, both of which are involved in cell motility [99,100].

Surprisingly, other studies have reported a role of EPAC in increasing VSMC proliferation and migration and promoting neointimal formation. Contradictory to the treatment with a PKA analog, treatment of rat aortic VSMCs with an EPAC activator significantly enhanced cell migration. In addition, overexpression of EPAC facilitated the development of neointimal formation in an ex vivo model [101]. Platelet-derived growth factor (PDGF) and basic fibroblast growth factor (bFGF) are both released by VSMCs and other cells present at the vascular injury site and contribute to cell migration and intimal thickening [102,103]. Kato et al. revealed that EPAC deficiency inhibits migration and attenuates neointimal formation by decreasing PDGF-induced intracellular Ca^2+^ elevation and suppressing cofilin-mediated lamellipodia formation [104]. The same group later reported that EPAC also contributes to bFGF-induced migration of VSMCs by activating the downstream signaling of PI3K/ protein kinase B (Akt) [105,106]. In a mouse carotid artery ligation model, the knockout of EPAC or its pharmacological inhibition significantly decreases neointimal formation by inhibiting VSMCs migration and proliferation [70]. Looking into the molecular mechanism of EPAC-mediated neointimal formation, EPAC increases the activity of PI3K/Akt signaling and induces mitochondrial fission and ROS production, both of which play a role in VSMCs activation during vascular remodeling [70,107,108].

**Figure 4:**
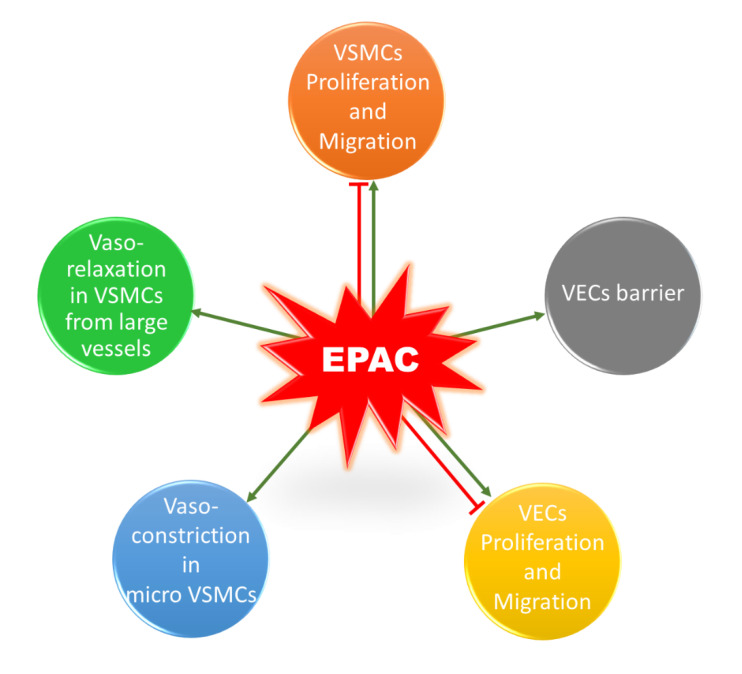
Role of EPAC in the vasculature. EPAC can promote or inhibit proliferation and migration in VSMCs. In addition, EPAC induces vasorelaxation in VSMCs extracted from large vessels, whereas it mediates cold-induced vasoconstriction in microVSMCs. EPAC has a controversial role in VECs; it can promote or inhibit cell proliferation and migration. EPAC also enhances VECs barrier and decreases permeability.

It is worth mentioning that a similar contradictory profile exists for the role of EPAC in vascular endothelial cells (VECs) (Figure 4). EPAC has been shown to differentially mediate adhesion of VECs isolated from microvessels and macrovessels [109]. In addition, EPAC and its effector, Rap1, enhance cell–cell contacts, mediating the vascular endothelial barrier and decreasing cell permeability [110,111]. Regarding the impact of EPAC on VEC proliferation and migration, some studies reported an inhibitory effect, while others showed that EPAC increases VEC proliferation and migration, promoting angiogenesis [112,113,114]. The discrepancy in these studies could arise from differences in model used and vascular beds from which VECs were obtained. Since this does not lie in the scope of our review, details for the role of EPAC in VECs can be found in other reviews [16,22,115].

## 4. Concluding Remarks

Although the role of EPAC in the vasculature is controversial, there is no doubt that it is a key player in the cardiovasculature. The reasons for this discordance partly lies in the differences between cellular model systems. Indeed, the susceptibility of VSMCs to phenotypic switching can be influenced by their site of origin and vascular bed from where the cells were isolated, in addition to differences in the species, strain, age and gender of the animal model [12].

Nevertheless, since EPAC plays a crucial role in vascular physiology and pathology, it could represent a potential target for drugs designed to treat atherosclerosis and hypertension. It is, therefore, important that future studies identify other downstream effectors that mediate Rap1-independent EPAC signaling to facilitate the development of new therapeutics that may target this family of cAMP effectors. Extensive research is also required to resolve the discrepancies in the role of EPAC in the vasculature and to translate in vitro and in vivo studies into clinical trials.
